# Biomechanical determinants of proficient 3-point shooters: markerless motion capture analysis

**DOI:** 10.3389/fspor.2026.1732293

**Published:** 2026-02-12

**Authors:** Dimitrije Cabarkapa, Damjana V. Cabarkapa, Andrew C. Fry

**Affiliations:** 1 Jayhawk Athletic Performance Laboratory – Wu Tsai Human Performance Alliance, Department of Health, Sport and Exercise Sciences, University of Kansas, Lawrence, KS, United States; 2 D2 Lab, Novi Sad, Serbia; 3 Fry Sports Performance, Lawrence, KS, United States

**Keywords:** basketball, coaching, motion capture, skill development, sport performance

## Abstract

Despite the growing recognition of 3-point proficiency as a defining element of modern basketball success, relatively few studies using markerless motion capture technology have examined the biomechanical characteristics that influence long-distance shooting effectiveness. Thus, the purpose of the present study was to investigate differences in biomechanical characteristics between proficient and non-proficient 3-point shooters during both the preparatory and release phases of the shooting motion. Twenty-four male basketball players participated in this study, of which eleven were classified as proficient (≥50%) and thirteen as non-proficient shooters (<50%). Following a standardized warm-up, each participant attempted ten non-consecutive 3-point shots (6.75 m) from the top of the key. Biomechanical data were collected using a three-dimensional markerless motion capture system operating at 120 Hz. Between-group differences were analyzed using Mann–Whitney U tests and independent t-tests, depending on weather the variable violated or met the assumption of normality (*p* < 0.05). Proficient shooters exhibited greater hip, knee, and ankle flexion (*g* > 1.694), resulting in a lower center of mass, as well as higher peak (*r* = 0.585) and mean hip angular velocities (*g* = 1.146) compared to their non-proficient counterparts during the preparatory phase of the shooting motion (large effect sizes). They also initiated the 3-point shooting motion with a wider stance, suggesting a more stable base, although stance alignment did not differ significantly between groups. No significant differences were observed in kinematic variables at the time point of the ball release, as both groups displayed similar values (e.g., jump height, release height). Collectively, these findings suggest that the success of a 3-point shot is primarily determined by biomechanical adjustments made during the preparatory phase, which provide the foundation for effective shot execution.

## 1 Introduction

Basketball is a fast-paced game that requires athletes, besides physiological preparedness, to possess a high level of skill, particularly shooting efficiency (1, 2). Over the past several decades, basketball has evolved dramatically, with increased emphasis on long-range scoring fundamentally altering offensive strategies at all levels of play. In the National Basketball Association (NBA), 3-point attempts have nearly doubled over the past decade, rising from approximately 22% to nearly 39% of all field-goal attempts in (2011 vs. 2021 season), while mid-range shots have declined substantially (3). This trend reflects broader strategic adjustments, as teams maximize scoring efficiency by prioritizing shots from beyond the arc, while minimizing mid-range attempts (4). Consequently, 3-point shooting efficiency has become a critical determinant of team success, regardless of the level of play (e.g., collegiate, professional), influencing game pacing, court spacing, and defensive schemes (3, 5, 6). Accordingly, the growing tactical reliance on efficient 3-point shooting highlights the need to identify the biomechanical and technical determinants of long-range shot execution in order to optimize individual technique and enhance on-court team performance.

To date, a considerable body of scientific literature has examined the biomechanical characteristics of free-throw and mid-range shooting motions, leaving the shooting performance beyond the arc (i.e., 3-point shot) vastly underexplored (7–14). For example, Ammar et al. (7) reported that among novice basketball players, greater knee flexion during the preparatory phase of the free-throw, followed by near-complete extension at ball release, was positively associated with successful outcomes, highlighting the role of efficient knee joint mechanics in shooting accuracy. Similarly, Cabarkapa et al. (11) found that proficient free-throw shooters (>70%) demonstrated greater knee, hip, and ankle flexion during the preparatory phase, which contributed to a lower elbow placement (i.e., due to center of mass being closer to the ground). With respect to release mechanics, Tran and Silverberg (14) identified an optimal free-throw release angle of approximately 52°, noting that smaller angles increase susceptibility to error. Release height has also been shown to play a critical role, as it is inversely related to release angle (15). Specifically, lower release heights require larger release angles, which ultimately increase the vertical displacement of the ball (15). Conversely, increasing release height allows players to adopt smaller release angles, thereby reducing the need for high movement velocities to achieve a successful shot (10).

Collectively, the aforementioned research reports provide valuable insight into the biomechanics of basketball shooting and emphasize the importance of consistent kinematic and kinetic sequencing in optimizing accuracy. However, most of this work has concentrated on free-throw or mid-range jump shots, which differ substantially from 3-point attempts in terms of distance and physical demands. The influence of shooting distance on kinematic and kinetic parameters has been well documented in the scientific literature. For instance, Okazaki and Rodacki (16) reported that as shooting distance increased, accuracy declined from 59% to 37% (6.4 m vs. 2.8 m), accompanied by reductions in release height and angle as well as compensatory increases in release velocity, highlighting critical movement adaptations that influence 3-point shooting performance. Consistent with these findings, Cabarkapa et al. (17) observed that while preparatory-phase mechanics remain stable between free throws and 2-point shots, 3-point shooting attempts required greater knee and hip flexion, lower elbow positioning, and a reduced release angle, underscoring the additional biomechanical adjustments necessary for long-range shooting. Taken together, this evidence demonstrates the distinct biomechanical demands of 3-point shooting and underscores the importance of further research to clarify how specific movement strategies contribute to proficiency at extended distances.

Despite the growing recognition of 3-point proficiency as a defining element of modern basketball success (3, 5, 18), relatively few studies have examined the biomechanical factors that distinguish proficient from non-proficient long-range shooters. Therefore, the purpose of the present study was to investigate differences in biomechanical characteristics between proficient and non-proficient 3-point shooters. Based on previous literature, it was hypothesized that significant between-group differences would be observed, with proficient shooters demonstrating greater lower-body flexion and more efficient release parameters compared to their non-proficient counterparts. These adaptations were expected to reflect patterns previously reported in free-throw and mid-range shooting motions, but to an even greater extent due to the increased distance and physical demands of the 3-point shot.

## 2 Materials and methods

### 2.1 Participants

Twenty-four male basketball players volunteered to participate in the present study, from which eleven were proficient (age = 22.6 ± 5.1 years; height = 181.5 ± 9.7 cm; body mass = 81.0 ± 11.3 kg) and thirteen non-proficient (age = 24.2 ± 8.4 years; height = 180.5 ± 6.7 cm; body mass = 79.9 ± 9.2 kg) 3-point shooters. Based on effect sizes reported in previously published research, a minimum sample size of *n* > 20 was required to detect a between-group difference with an alpha level of 0.05 and a desired statistical power of 0.80 (GPower, version 3.1, Germany). Inclusion criteria required a minimum of five years of organized basketball playing experience (e.g., high school, collegiate) and current participation in basketball-related training for at least six months (≥2 sessions per week). Exclusion criteria included any upper or lower-extremity injury within the previous six months, a history of major orthopedic surgery, or any functional limitations or pain that could impair full joint range of motion and alter shooting technique. Also, all participants were right-handed and regularly participated in basketball-related training activities more than three times per week. The testing procedures performed in this study were previously approved by the University of Kansas Institutional Review Board (No. 00149119) and were conducted in accordance with the Declaration of Helsinki. All participants provided written informed consent prior to participation.

### 2.2 Procedures

Before the start of the testing procedures, each participant completed a standardized warm-up protocol (10 min) consisting of dynamic stretching exercises (e.g., high knees, A-skips, walking lunges, quad pulls, butt kicks), followed by 10–15 practice shots from self-selected positions on the court (19). Then, each participant attempted ten non-consecutive 3-point shots (6.75 m) on the top of the key (i.e., shoulders facing directly to the basket). All shots were performed from a stationary position without a preparatory step or approach, with athletes initiating the shot from a set stance. The basket height (305 cm) and ball size (74.93 cm) adhered to National Collegiate Athletic Association regulation standards. To prevent fatigue from influencing 3-point shooter performance, a rest period of 10–15 s was given between each shooting attempt. Three research assistants were present throughout the testing procedures to complete rebounding and passing tasks. Also, to avoid possible distractions, all participants completed the testing procedures individually with no audience.

**Figure 1:**
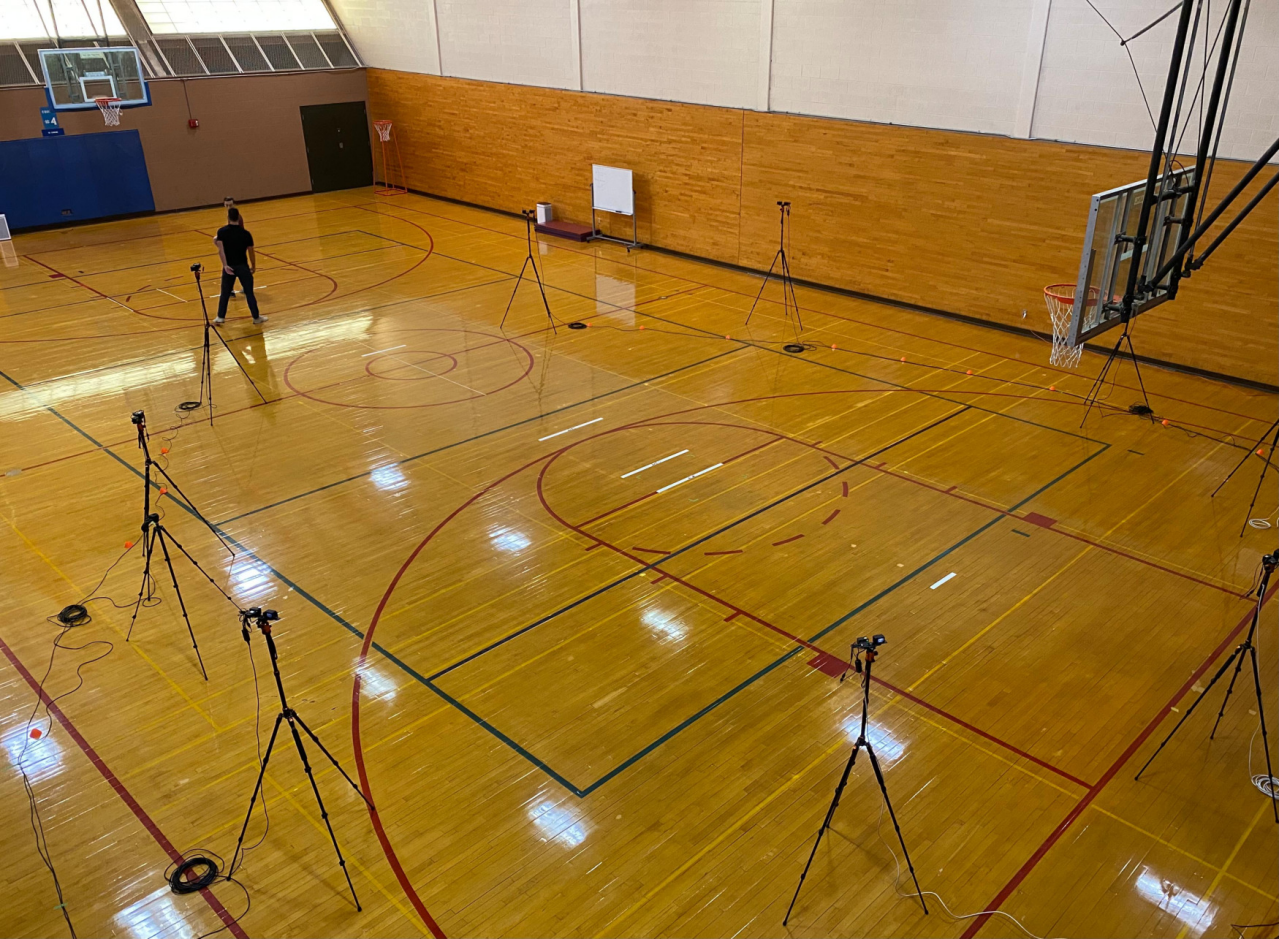
A photo of the testing setup, including a markerless motion capture system.

Biomechanical data were collected via an innovative three-dimensional markerless motion capture system (SwRI Enable®, San Antonio, TX, USA), which incorporated nine RXO-II high-definition cameras (Sony Corporation, Tokyo, Japan). This technology has been shown to be a valid and reliable tool for evaluating various types of human movement, including basketball shooting mechanics (19–21). These cameras were systematically arranged and synchronized to provide full coverage of the half-court area, while allowing participants to freely move around the court without any restrictions. The system operated at a sampling rate of 120 Hz and was calibrated based on the manufacturer's instructions prior to the data collection process. An illustration of the testing setup is provided in Figure 1.

Biomechanical variables analyzed in this study were selected based on previously published research reports (7, 9, 11, 15, 16, 19, 22). The preparatory phase (PP) was defined as the initial concentric movement within the 3-point shooting sequence (i.e., initial upward movement), while the release phase (RP) corresponded to the moment the ball left the shooter's hand. The average value across ten shooting attempts was used for performance analysis purposes. A description of each kinetic and kinematic parameter can be found in Table 1.

**Table 1:** Definition of the dependent variables examined in the present study.

Variable [unit]	Definition
Knee angle [deg]	Internal angle between the thigh and shank.
Hip angle [deg]	Internal angle between the torso and the thigh.
Ankle angle [deg]	Internal angle between the shank and the foot.
Elbow angle [deg]	Internal angle between the upper arm and forearm.
Center of mass height [ratio]	Perpendicular distance between the point within the body at which the overall shooter's mass is located and the ground adjusted by the participant's height.
Stance width [cm]	Distance between the placement of the right and left foot during the preparatory phase of the shooting motion.
Stance alignment [cm]	Vertical feet alignment during the preparatory phase of the shooting motion. A positive value indicates that the right foot is placed more forward than the right foot.
Elbow height [ratio]	Perpendicular distance between the olecranon process and the ground during the preparatory phase of the shooting motion divided by the participant's height.
Release height [ratio]	Perpendicular distance between the hand and the ground at the time point of the ball release divided by the participant's height.
Jump height [cm]	Perpendicular distance between the heel (calcaneus) and the ground at the time point of the ball release.
Forearm angle [deg]	Angle between the forearm and an imaginary vertical axis. A positive value represents the amount of lateral elbow deviation.
Transition time [sec]	The time elapsed between start of preparatory and start of release phase of the shooting motion.
Range of motion [deg]	The amount of movement that occurred in each joint between the preparatory to the release phases of the shooting motion.
Mean velocity [deg/s]	The rate of change in angular displacement observed in the specific joint between the preparatory and the release phases of the shooting motion.
Peak velocity [deg/s]	The maximal angular velocity observed in the specific joint between the preparatory and the release phases of the shooting motion.

### 2.3 Statistical analysis

Shapiro–Wilk tests were used to examine the assumption of normality. If the assumption of normality was met, independent *t*-tests (mean and standard deviation) were used to examine statistically significant between-group differences (i.e., proficient vs. non-proficient), using Hedges' g to depict the effect size magnitude (i.e., g < 0.20 small; g = 0.20–0.79 moderate; g > 0.80 large) (23). Conversely, if the assumption of normality was violated, Mann–Whitney U-test (median and interquartile range) were used to examine statistically significant between-group differences. The effect sizes were calculated by dividing the Z-statistic with the square root of the sample size (i.e., r = Z/√N; r < 0.30 small; r = 0.30–0.50 moderate; r > 0.50 large) (24). Based on previously published research reports and the level of playing experience, participants who made ≥50% of their shots were classified as proficient, and the ones that made <50% were classified as non-proficient (25). The *α* level of *p* < 0.05 was used as a criterion for statistical significance. All statistical analysis procedures were completed in SPSS® (Version 28.0; Chicago, IL, USA).

## 3 Results

Descriptive data for each dependent variable (mean and standard deviation or median and interquartile ranges) and their respective statistical inferences are presented in Table 2. The average 3-point shooting accuracy was 58.3 ± 9.2% for proficient (≥50%) and 25.3 ± 6.6% for non-proficient (<50%) shooters.

**Table 2:** Descriptive statistics, mean (standard deviation) or median (interquartile range), and statistical comparisons between proficient (≥50%) and non-proficient (<50%) 3-point shooters.

Variable [unit]	Non-proficient	Proficient	*p*-value	ES
Knee angle – PP [deg]^*^	113.2 (4.2)	94.3 (5.4)	**<0**.**001**	3.961 (L)
Knee angle – RP [deg]^*^	172.2 (5.3)	164.9 (7.3)	**0**.**010**	1.161 (L)
Knee ROM [deg]^*^	58.9 (4.7)	70.5 (6.7)	**<0**.**001**	2.036 (L)
Knee peak velocity [deg/s]	378.4 (35.0)	397.1 (67.4)	0.393	0.357 (S-M)
Knee mean velocity [deg/s]	196.8 (48.1)	210.4 (37.6)	0.454	0.311 (S-M)
Hip angle – PP [deg]^*^	155.9 (7.6)	143.1 (7.5)	**<0**.**001**	1.694 (L)
Hip angle – RP [deg]^#^	174.8 (8.3)	172.0 (1.7)	0.252	0.241 (S)
Hip ROM [deg]^*^	16.5 (3.6)	27.9 (7.6)	**<0**.**001**	1.975 (L)
Hip peak velocity [deg/s]^#*^	145.5 (53.5)	214.5 (87.7)	**0**.**003**	0.585 (L)
Hip mean velocity [deg/s]^*^	54.3 (22.9)	86.6 (33.4)	**0**.**015**	1.146 (L)
Ankle angle – PP [deg]^#*^	62.3 (14.4)	50.1 (9.4)	**<0**.**001**	0.655 (L)
Ankle angle – RP [deg]	118.3 (11.9)	117.2 (12.8)	0.829	0.089 (S)
Ankle ROM [deg]	53.2 (19.1)	64.9 (13.6)	0.009	0.695 (M-L)
Ankle peak velocity [deg/s]	449.2 (122.5)	487.3 (69.1)	0.371	0.374 (S-M)
Ankle mean velocity [deg/s]	175.6 (77.6)	189.0 (61.8)	0.548	0.189 (S)
Elbow angle – PP [deg]	84.4 (15.5)	81.9 (15.9)	0.706	0.159 (S)
Elbow angle – RP [deg]^#^	158.8 (2.7)	159.3 (1.8)	0.228	0.245 (S)
Elbow ROM [deg]	73.1 (13.5)	77.8 (15.2)	0.429	0.329 (S-M)
Elbow peak velocity [deg/s]	1,006.9 (149.0)	1,070.2 (136.8)	0.293	0.441 (S-M)
Elbow mean velocity [deg/s]	258.9 (83.2)	247.6 (70.7)	0.725	0.145 (S)
COM height – PP [ratio]*	0.56 (0.03)	0.49 (0.02)	**<0**.**001**	2.698 (L)
COM height – RP [ratio]	0.71 (0.04)	0.72 (0.04)	0.289	0.250 (S-M)
COM change [ratio]^*^	0.15 (0.06)	0.22 (0.04)	**0**.**003**	1.349 (L)
COM – peak velocity [m/s]	1.69 (0.46)	1.84 (0.33)	0.362	0.369 (S-M)
COM – mean velocity [m/s]	0.94 (0.38)	1.23 (0.29)	0.094	0.847 (L)
Transition time [sec]^#^	0.35 (0.16)	0.36 (0.10)	0.361	0.196 (S)
Stance width – PP [cm]^*^	27.4 (7.8)	34.3 (4.2)	**0**.**048**	1.075 (L)
Stance alignment – PP [cm]	8.7 (7.1)	12.9 (7.9)	0.306	0.556 (M)
Elbow height – PP [ratio]^*^	0.64 (0.07)	0.57 (0.06)	**0**.**042**	1.066 (L)
Release height [ratio]^#^	1.19 (0.13)	1.20 (0.13)	0.303	0.218 (S)
Vertical jump height [cm]	23.7 (4.7)	25.8 (6.4)	0.376	0.379 (S-M)
Forearm angle – PP [deg]	33.7 (12.1)	34.4 (7.1)	0.790	0.069 (S)
Forearm angle – RP [deg]	13.4 (6.9)	12.8 (5.0)	0.823	0.098 (S)

ES – effect size (L – large; M – moderate; S – small); PP – preparatory phase; RP – release phase; ROM – range of motion; COM – center of mass; (#) non-normally distributed variable; (*) – statistically significant between-group differences.

Bold values represent statistically significant difference (*p* < 0.05).

Significantly smaller knee angles at both the preparatory and release phases of the shooting motion and greater knee joint range of motion were observed within the proficient group of 3-point shooters when compared to their non-proficient counterparts. In a similar manner, proficient shooters exhibited a smaller hip angle at the preparatory phase of the shooting motion and greater range of motion in the hip joint, while no between-group differences were noted in hip angle at the time point of the ball release. Also, both peak and mean hip angular velocities were greater in proficient than non-proficient shooters.

The ankle angle at the preparatory phase of the shooting motion was notably smaller in proficient than non-proficient group of 3-point shooters, indicating greater ankle joint flexion. Besides, the center of mass and elbow height (relative to the participant's stature) were lower and the change in center of mass height was greater within the group of proficient than non-proficient shooters. In addition, proficient shooters demonstrated greater stance width at the preparatory phase of the shooting motion, while no between-group differences were observed in stance alignment.

No differences in any other biomechanical parameters of interest between the proficient and non-proficient group of 3-point shooters were observed (e.g., forearm angle, ankle and knee peak and mean velocities, forearm angle, transition time, center of mass peak and mean velocities, vertical jump height), and all were relatively small to moderate in the effect size magnitude. In addition, no between-group differences were observed in age (*p* = 0.486), height (*p* = 0.959), and body mass (*p* = 0.826).

## 4 Discussion

The purpose of the present study was to examine biomechanical differences between proficient and non-proficient 3-point shooters. To the best of our knowledge, this is one of the first investigation to address these differences using an innovative markerless motion capture system, providing a non-invasive and time-efficient approach to movement analysis. The results support the hypothesis that kinematic adjustments in the lower-body during the preparatory phase of the shooting motion are critical for achieving higher 3-point shooting accuracy and serve as distinguishing characteristics of proficient shooters. Specifically, proficient shooters displayed greater flexion at the hip, knee, and ankle joints during the preparatory phase of the shooting motion when compared to their non-proficient counterparts, which ultimately lowered their center of mass, along with higher peak and mean hip angular velocities. They also initiated the shooting motion with a wider stance, suggesting a more stable base, although stance alignment did not differ between groups. Notably, no significant differences were observed in any kinematic variables at the time point of the ball release (i.e., release phase), as both groups displayed similar values. In essence, the findings of the present study indicate that the success of a 3-point shot is largely determined by multiple factors occurring before the ball release, with the preparatory phase mechanics establishing the foundation for the eventual outcome.

When examining the preparatory phase of the 3-point shooting motion, the findings of the present study align with previous research on the free-throw (7, 11, 26). Specifically, greater flexion in the knee, hip, and ankle joints, observed within a group of proficient shooters, ultimately caused the lower center of mass, which is consistent with the results obtained by Cabarkapa et al. (11). This adjustment is also accompanied by a lower elbow positioning relative to the shooter's stature, further reinforcing the importance of coordinated lower and upper-body mechanics (15). However, while the trends were similar, the magnitudes differed slightly. For instance, Cabarkapa et al. (11) reported knee and ankle angles of 101.1° and 52.6° among proficient free-throw shooters, whereas the results obtained in this study revealed greater flexion magnitudes, with knee and ankle angles of 94.3° and 50.1°, respectively. Considering that 3-point shot requires increased lower-body force and power production due to an increase in shooting distance compared to free-throw shot (6.75 m vs. 4.57 m), these biomechanical adjustments are expected to help offset an increase in shooting distance. Nakano et al. (27) further support this interpretation, showing that joint work in the lower-limbs significantly increases when shooting from longer distances (e.g., 3-point shot), emphasizing the need for sufficient energy transfer from the legs to the shooting arm in order to maintain consistent upper-body mechanics. Similarly, the findings of the present study provide additional confirmation, as not only was elbow position relative to the shooter's body height lower in proficient than in non-proficient shooters, but it was also lower in 3-point shooting compared to the free-throw (0.57 vs. 0.68), respectively (11).

Nonetheless, while increased lower-body flexion (e.g., knee and hip joints) is important for generating the necessary force and power, excessive flexion may be counterproductive (28). For example, Ammar et al. (7) reported that novice basketball players who displayed exaggerated knee flexion in the preparatory phase of the free-throw shooting motion experienced a decline in shooting accuracy. In contrast, proficient shooters seem to demonstrate greater, yet controlled ranges of motion, as evidenced by the data obtained in the present study. Supporting this, Irawan et al. (26) found that knee angle at the release phase of the 3-point shooting motion was nearly identical to the results of this investigation (i.e., 164.3° vs. 164.9°). Hence, confirming that the range of motion primarily increased with the changes within the preparatory phase, rather than the release phase. This observation also aligns with research findings obtained by Franca et al. (29), who demonstrated that ball release typically occurs prior to full joint extension at the peak of the jump. In this study, the knee angle at the time point of the ball release was 164.9°, short of full extension (180°), indicating that the critical adjustments influencing 3-point shot success tend to occur earlier, during the preparatory phase of the shooting motion. Still, the release phase should not be overlooked. Instead of assuming its lack of importance, it is more accurate to note that all participants in the present study appeared to reach adequate release conditions (e.g., hip, knee, and ankle angles). Thus, while the release phase represents a stable endpoint, the lower-body mechanics within the preparatory phase of the shooting motion remains the key determinant of superior 3-point shooting proficiency.

Another interesting finding in the present study was the considerably greater peak and mean hip angular velocities observed in proficient compared to non-proficient 3-point shooters. Greater flexion in the hips, knees, and ankles during the preparatory phase of the shooting motion likely allows proficient 3-point shooters to produce a faster and more explosive extension, ultimately resulting in higher hip velocities. Jessop and Pain (30) provide support for this idea, as their findings indicate that greater joint flexion can contribute to higher angular velocities, particularly when proximal segments are involved (e.g., hips, shoulders). Although further research is needed to confirm these mechanisms, non-proficient shooters may display less coordinated and slower movement patterns, which in turn could limit and impair hip angular velocity (15). Interestingly, while only the hip angular velocity differences reached statistical significance, both mean and peak values at the knee, ankle, and center of mass showed similar trends toward greater values being observed in proficient compared to non-proficient 3-point shooters, with small-to-moderate effect size magnitudes. This suggests that greater hip velocity reflects more forceful and coordinated hip extension during the upward propulsive phase of the 3-point shooting motion, thereby facilitating vertical force generation and efficient energy transfer through the trunk, shoulders, and arms into the ball (15, 22). Although force-producing capabilities were not directly measured in this investigation, it is reasonable to assume that increasing shooting distance requires players to attain greater force and power producing capabilities (31, 32). Accordingly, increases in hip velocity, together with the need for greater force production required for long-distance shots, ultimately contribute to the higher power outputs necessary to propel the ball toward the basket (33). Furthermore, it is interesting to note that the velocity magnitudes across all analyzed joints (i.e., ankle, hip, knee) were greater in the present study compared to those reported during free-throw shooting (19), further emphasizing the increased lower-body force and power demands necessary to execute longer-range shots.

In addition to examining the kinematics of the preparatory phase of the shooting motion, it is important to consider foot alignment (i.e., forward-backward positioning) and stance width (22). A common coaching cue is to initiate the shooting motion with the feet approximately shoulder-width apart, while positioning the foot on the same side as the shooting hand slightly ahead, about half a foot length in front of the other (34). Placing the shooting-side foot forward provides a more stable base and helps limit excessive rotation of the shoulders, trunk, and pelvis during release, thereby optimizing the efficiency of the shooting motion (15, 22). The findings of the present investigation seem to be consistent with the aforementioned recommendations, as both proficient and non-proficient shooters tended to position the right foot in front of the left, with positive values indicating greater forward displacement of the right foot. Although between-group differences were not statistically significant, proficient shooters exhibited a more pronounced staggered stance than non-proficient shooters (12.9 cm vs. 8.7 cm), further emphasizing the potential importance of this biomechanical adjustment. Additionally, it should be noted that stance width was considerably greater in proficient than non-proficient shooters, suggesting an adaptation to improve stability and meet the increased force and power-producing demands of the 3-point shot. Compared to the stance width magnitudes observed in free-throw shooting motion, the results obtained in the present investigation showed slightly greater values for the 3-point shot, which is expected given the greater stability required as player moves further away from the basket (19, 35). Overall, adopting a wider and slightly staggered stance appears to reduce forward-backward sway during the release phase of the shooting motion and facilitates smoother force transfer from the lower-body through the kinetic chain, resulting in more consistent shooting mechanics (15, 35). Moreover, this stance helps maintain a direct line between the shooting arm, the ball, and the basket, thereby improving accuracy and reducing the need for compensatory adjustments (15, 22).

When examining the release phase of the 3-point shooting motion, no statistically significant differences were found between proficient and non-proficient 3-point shooters, with all effect sizes ranging from trivial to small. Although prior research highlights the critical importance of release parameters for shooting success (11, 14, 36), the present study found that vertical jump height and ball release height relative to player stature were nearly identical between groups (1.20 vs. 1.19). These values align with recent reports among proficient 3-point shooters, where heel height (i.e., proxy for jump height) averaged 28.9 cm (11). Thus, rather than suggesting that release conditions are irrelevant, these results indicate that both proficient and non-proficient shooters attained adequate release mechanics, while the decisive differences resided in the preparatory phase of the shooting motion, which ultimately influenced shooting accuracy. Furthermore, although proficient shooters displayed greater hip, knee, and ankle flexion during the preparatory phase of the shooting motion, no differences were observed at the transition time from the start of the shooting motion to the time point of ball release. This suggests that proficient 3-point shooters achieved the necessary biomechanical adjustments without requiring additional time, underscoring that the timing of shot execution had minimal impact.

While this study provides valuable insights into the biomechanical characteristics of the 3-point shooting motion within both preparatory and release phases, several limitations should be acknowledged. First, the sample was homogeneous, consisting exclusively of male basketball players, and did not include athletes competing across different levels of play (e.g., professional, collegiate). Additionally, the sample size was relatively small, which may limit the generalizability of the findings. Second, playing positional differences were not considered (e.g., centers, forwards, guards), even though variations in anthropometrics across positions could influence shooting mechanics. Finally, the experimental design did not account for the presence of a defender, which has been shown to alter shooting execution (37). Therefore, future research is warranted to determine whether the biomechanical parameters observed in the present study (e.g., knee angle, hip angular velocity, stance alignment, transition time) remain consistent under defensive pressure and in more competitive game-like environments.

In conclusion, the findings of the present study indicate that kinematic adjustments in the lower body during the preparatory phase of the shooting motion are critical for achieving higher 3-point shooting accuracy and represent key distinguishing features of proficient shooters (≥50%). Specifically, proficient shooters demonstrated greater hip, knee, and ankle flexion, which lowered their center of mass, when compared to their non-proficient counterparts (<50%) as well as higher peak and mean hip angular velocities. Also, proficient shooters initiated the 3-point shooting motion with a wider stance, suggesting a more stable base, although stance alignment did not differ between groups. Importantly, no significant differences were observed in kinematic variables at the ball release, as both groups displayed similar values. Collectively, these results suggest that the success of a 3-point shot is primarily determined by biomechanical adjustments made during the preparatory phase, which establish the foundation for effective shot execution.

## Data Availability

The datasets presented in this article are not readily available because of University's Institutional Review Board rules and regulations. Requests to access the datasets should be directed to dcabarkapa@ku.edu.
